# The First Clinical Renal Xenotransplantation Study (EXPAND): Evaluating Safety, Function, and Zoonotic Risks

**DOI:** 10.1002/hcs2.70044

**Published:** 2025-12-04

**Authors:** Björn Nashan

**Affiliations:** ^1^ Research Centre of Big Data and Artificial Intelligence of Medicine The First Affiliated Hospital of Sun Yat‐Sen University Guangzhou China; ^2^ First Affiliated Hospital, School of Life Sciences and Medical Center, The Transplantation Center University of Sciences & Technology of China Hefei Anhui China

**Abbreviations**
BLA: Biologics License Application
EPO: erythropoietin
ESRD: end‐stage renal disease
FDA: Food and Drug Administration
NHP: nonhuman primate
NYU: New York University
pCMV: porcine cytomegalovirus

On November 3, 2025, United Therapeutics Corporation (Nasdaq: UTHR) announced the first clinical xenotransplantation within its EXPAND study, involving transplantation of the UKidney into a patient with end‐stage renal disease (ESRD) at New York University (NYU) Langone Health. The UKidney is a genetically engineered porcine kidney incorporating 10 gene modifications—6 human gene insertions to enhance immunologic compatibility and 4 porcine gene knockouts to minimize rejection risk and control graft growth [1]. In the context of the first xenotransplantation studies, “EXPAND” refers to the US Food and Drug Administration (FDA) “expanded access” program, often colloquially known as compassionate use. This program provides a pathway for seriously ill patients who have no other treatment options to receive an investigational medical device, in this case, a genetically modified pig heart, outside of a traditional clinical trial.

This announcement arrived just in time, since the last recipient, a 67‐year‐old male recipient at Massachusetts General Hospital, underwent removal of a genetically modified porcine kidney 9 months post‐transplant due to progressive graft dysfunction in October 2025. The patient survived 271 days, representing the longest reported survival following a pig‐to‐human kidney xenotransplantation. Previous recipients experienced early graft loss or rejection. The case demonstrates extended graft viability and provides valuable clinical insight into the long‐term outcomes of renal xenotransplantation [2].

The EXPAND study (NCT06878560) is a multicenter, open‐label, interventional clinical trial sponsored by United Therapeutics Corporation. The study represents the first regulated clinical xenotransplantation program evaluating the *UKidney*, a 10‐gene–edited porcine kidney in patients with ESRD who are ineligible for or unlikely to receive a human donor kidney within 5 years. The first xenotransplantation under this protocol was successfully performed at NYU Langone Health on November 3, 2025 [3, 4].

The *UKidney* is derived from a genetically engineered pig designed to enhance immunological compatibility and control organ growth. The genome modification includes six human gene insertions (knock‐ins) that promote immune tolerance, regulate coagulation, and protect against complement‐mediated injury, as well as four porcine gene deletions (knockouts) that remove carbohydrate epitopes responsible for hyperacute rejection and prevent excessive graft growth (Table 1) [5].

**Table 1:** Genetic modifications in the donor pig (10 GE Xenokidney) [5].

Type	Gene	Full name/Description	Function/Purpose	Intended effect
Knockout (disabled pig genes)	*GGTA1*	α‐1,3‐Galactosyltransferase	Enzyme adding α‐Gal carbohydrate epitopes	Prevents hyperacute rejection caused by anti‐Gal antibodies
	*B4GALNT2*	β‐1,4‐N‐acetyl‐galactosaminyltransferase 2	Enzyme adding carbohydrate structures	Reduces immune recognition of pig antigens
	*CMAH*	Cytidine monophosphate‐N‐acetylneuraminic acid hydroxylase	Produces Neu5Gc, a nonhuman sialic acid	Prevents antibody‐mediated rejection to Neu5Gc
	*GHR*	Growth Hormone Receptor	Hormone receptor controlling organ growth	Prevents excessive kidney growth in recipient
Knock‐in (added human genes)	*CD55 (DAF)*	Decay‐Accelerating Factor	Complement regulatory protein	Inhibits complement activation; reduces hyperacute rejection
	*CD46*	Membrane Cofactor Protein	Complement regulatory protein	Protects graft from antibody‐mediated complement damage
	*TBM (Thrombomodulin)*	Thrombomodulin	Coagulation regulatory protein	Prevents microthrombi and thrombotic microangiopathy
	*EPCR*	Endothelial Protein C Receptor	Coagulation regulatory protein	Reduces coagulation and inflammatory injury; enhances graft survival
	*HO1 (Heme Oxygenase‐1)*	Heme Oxygenase‐1	Anti‐oxidative, anti‐apoptotic, anti‐inflammatory enzyme	Reduces oxidative stress, inflammation, and cell death in graft
	*CD47*	Cluster of Differentiation 47	“Don't eat me” signal molecule	Suppresses macrophage activation and T‐cell infiltration

The primary objective of EXPAND is to evaluate the safety, tolerability, and functional performance of the xenograft at 24 weeks post‐transplantation. Primary endpoints include patient and graft survival, while secondary endpoints assess glomerular filtration rate, proteinuria, and the incidence of xenograft rejection, infection, or other adverse events. Long‐term follow‐up will monitor durability of graft function, immune adaptation, and potential zoonotic risks (Table 2) [4].

**Table 2:** EXPAND Study – Evaluation of the 10‐Gene‐Edited UKidney in End‐Stage Renal Disease (ESRD) – Short summary [4].

Category	Details
Study title	A Study to Evaluate the Safety and Efficacy of the 10‐Gene‐Edited UKidney in &QJ0;Patients with End‐Stage Renal Disease
ClinicalTrials.gov Identifier	NCT06878560
Sponsor	United Therapeutics Corporation
Study type	Interventional (Clinical Trial), open‐label, multicenter
Study objective	To assess the safety, tolerability, and preliminary efficacy of xenotransplantation using a 10‐gene‐edited porcine kidney (UKidney) in patients with ESRD
Study design	Phase 1/2/3 trial; single‐arm; estimated 6 participants in the initial cohort; expansion planned up to ~50 patients pending safety review
Intervention	Transplantation of a genetically modified porcine kidney (UKidney) containing 10 genetic edits: 6 human gene insertions (knock‐ins) and 4 porcine gene deletions (knockouts)
Primary endpoint (24 weeks)	– Graft survival and function – Patient survival – Incidence of acute xenograft rejection
Secondary endpoints	– Changes in glomerular filtration rate (GFR) – Proteinuria – Adverse events (including zoonotic and opportunistic infections) – Quality of life (QoL) assessments
Follow‐up period	Primary endpoint at 24 weeks; long‐term follow‐up for patient and graft survival up to several years
Population	Adult patients 55–70 years (adult, older adult) with ESRD who are not eligible for or unlikely to receive a human donor kidney within 5 years
Key inclusion criteria	– Diagnosed ESRD – No contraindication to surgery or immunosuppression – Ability to provide informed consent
Key exclusion criteria	– Active infection – Uncontrolled comorbidities – Prior xenotransplantation exposure – Contraindications to immunosuppression
Primary sites	NYU Langone Health and additional U.S. transplant centers (future expansion planned)
Estimated start date	November 2025
Status	Recruiting/Early Phase (first patient transplanted November 2025)

The trial is structured as a Phase 1/2/3 study with an initial cohort of six recipients undergoing xenotransplantation at select U.S. centers, followed by planned expansion to approximately 50 participants pending favorable safety review. Data from EXPAND are expected to inform regulatory strategies toward a Biologics License Application (BLA) and contribute to the development of standardized safety and efficacy frameworks for clinical renal xenotransplantation [4].

With the major immunological barriers—namely, hyperacute and acute rejection—now largely controlled based on prior experience [6], current research efforts have shifted toward evaluating functional graft performance, particularly glomerular function. A key challenge may be proteinuria, as therapeutic monoclonal antibodies that modulate complement‐mediated immune responses can be lost through filtration, a phenomenon previously observed in nonhuman primate (NHP) models [7]. Another critical focus is the potential transmission of zoonotic agents. While NHP studies have not demonstrated transmission of porcine endogenous retroviruses, porcine cytomegalovirus (pCMV) remains a specific concern in xenotransplantation [8]. Although pCMV is not believed to infect humans or NHPs, viral replication can occur in graft tissue from CMV‐positive donor pigs under intense recipient immunosuppression. pCMV replication has been detected in recipients of infected kidney xenografts and is often associated with early nuclear dystrophia in graft cells, suggesting a role in early graft dysfunction and rejection. Notably, the first heart xenotransplant recipient succumbed to complications associated with pCMV infection, as it could not be excluded that the pCMV‐positive organ elicited a human immune response [9, 10].

Normal physiological parameters differ substantially between pigs and humans, with some variations holding potential clinical significance for renal xenotransplantation, which will now be evaluated in the setting of a clinical study. While electrolytes such as sodium, potassium, and bicarbonate show minimal divergence, marked differences exist in hemoglobin, phosphate, and albumin levels. Lower hemoglobin in pigs may affect erythropoietin (EPO) regulation post‐transplant, while elevated serum phosphate could disrupt bone and mineral metabolism via altered parathyroid hormone, vitamin D, and fibroblast growth factor 23 signaling. Reduced albumin concentrations in pigs may influence glomerular filtration dynamics when exposed to higher human albumin levels. Although therapeutic measures could address several of these disparities, the lack of interaction between porcine renin and human angiotensinogen represents a significant physiological barrier. NHP studies have reported hypovolemia, hypotension, and transient renal dysfunction without histologic rejection, likely due to impaired renin–angiotensin–aldosterone system activity or altered renal responsiveness to antidiuretic hormone. Additionally, post‐transplant anemia may result from inadequate stimulation of host erythropoiesis by porcine EPO, compounding the challenges of functional integration across species [11].

The EXPAND study is expected to generate the first robust human data on genetically engineered renal xenografts, defining their capacity to sustain metabolic and hemodynamic stability in human recipients. It will also clarify unresolved issues such as protein loss, pCMV activation, and cross‐species hormonal and renal regulation. Its outcomes are likely to shape future immunologic protocols, biosafety standards, and ethical frameworks—positioning xenotransplantation as a potential long‐term solution to the global organ shortage.

Moreover, the study ultimately aims to address one of the most pressing challenges in modern medicine—the global shortage of donor organs for patients with end‐stage organ failure. While renal transplantation benefits from alternative replacement options such as dialysis, patients awaiting heart, liver, or lung transplantation have no comparable life‐sustaining therapies available. By advancing xenotransplantation from experimental, compassionate‐use interventions to a structured clinical trial framework, the EXPAND study represents a critical step toward developing a sustainable, scalable source of transplantable organs across multiple disciplines [10, 12, 13, 14, 15, 16, 17].

In addition, this trial helps bridge the gap between preclinical feasibility and clinical implementation by establishing a model for ethically and scientifically governed xenotransplantation research. It introduces rigorous oversight through professional consensus, regulatory supervision, and standardized clinical protocols, ensuring both scientific integrity and patient safety. Equally important, it integrates public engagement as a central element—acknowledging that societal understanding and acceptance will be essential for legitimizing xenotransplantation as a viable, life‐saving therapeutic option in the future [18, 19, 20].

## Author Contributions

**Björn Nashan:** conceptualization, methodology, writing – review and editing, writing – original draft, validation.

## Ethics Statement

The author have nothing to report.

## Consent

The author have nothing to report.

## Conflicts of Interest

Professor Björn Nashan is a member of the *Health Care Science* Editorial Board. To minimize bias, he was excluded from all editorial decision‐making related to the acceptance of this article for publication.

## Data Availability

The author have nothing to report.
